# Case Report: Sudden cardiac death due to ventricular myocardial non-compaction

**DOI:** 10.12688/f1000research.24583.1

**Published:** 2020-08-26

**Authors:** Syrine Mannoubi, Med Amin Mesrati, Ibn Hadj Amor Hassen, Taha Hasnaoui, Hiba Limem, Marwa Boussaid, Nouha Ben Abdejlil, Abir Aissaoui

**Affiliations:** 1 Department of Forensic Medicine, Taher Sfar Hospital, University of Monastir, Mahdia, 5100, Tunisia; 2 Department of Cardiology, Taher Sfar Hospital, University of Monastir, Mahdia, 5100, Tunisia; 3 Department of Pathology, Fattouma Bourguiba Hospital, University of Monastir, Mahdia, 5100, Tunisia

**Keywords:** Isolated Non compaction of the Ventricular Myocardium, Cardiomyopathy, Histology, Sudden Cardiac Death, Autopsy.

## Abstract

Ventricular non-compaction (VNC) is a rare myocardium disorder, which can be both genetic and sporadic. A poor wall compaction process or an excessive trabeculae formation may be at the genesis of myocardial hypertrabeculation with multiple recesses. It is often complicated by ventricular dysfunction, arrhythmias and cardiac embolism. Herein we report a case of a 20-year-old male patient with no particular past medical history who was followed up at the cardiology department for dyspnea. Echocardiography showed reduced ejection fraction of the left ventricle with potential hypertrabeculation in the right ventricle, confirmed by cardiac MRI. The patient was not put under medication and was later lost to follow-up. He died few months later without a clear cause explaining death. A forensic autopsy was performed that attributed death to acute ventricle arrhythmia secondary to VNC, emphasizing the major role of an early and specific treatment to avoid such a fatal outcome.

## Introduction

Ventricular non-compaction (VNC) is a complex and heterogeneous cardiomyopathy first described in 1926. It is a rare disease with a reported prevalence of 0.014–0.17% ^1^. Several terms are used to describe this disease namely: “cardiac hyper and excessive trabecularization”, “spongy myocardium”, “honeycomb myocardium”, or “persisting myocardial sinusoids”, and even “isolated ventricular abnormal trabeculation”. It is characterized by hypertrabeculation with an excessive lace-like network of trabeculae and deep trabecular pockets in the ventricle, creating a perfect environment for thrombi formation. VNC can be detected in all age groups, ranging from the fetal period to adulthood ^2^. It can occur sporadically or is hereditary secondary to chromosomal abnormalities. It can also be associated with other cardiac diseases, which may be congenital. Additionally, VNC is represented by a large spectrum of symptoms and clinical features ranging from normal variants to pathological phenotypes. Indeed, VNC may remain asymptomatic until a complication occurs. Cardiologists must pay more attention to various clinical manifestations, including heart failure, arrhythmias and cardio embolic events, which can be related to VNC, to initiate an early treatment and avoid potentially fatal complications ^3^.

Herein, we report a fatal case of VNC in a 20-year-old male, attested by the autopsy, and we discuss different mechanisms involved in the occurrence of death.

## Case report

**Figure 1.**
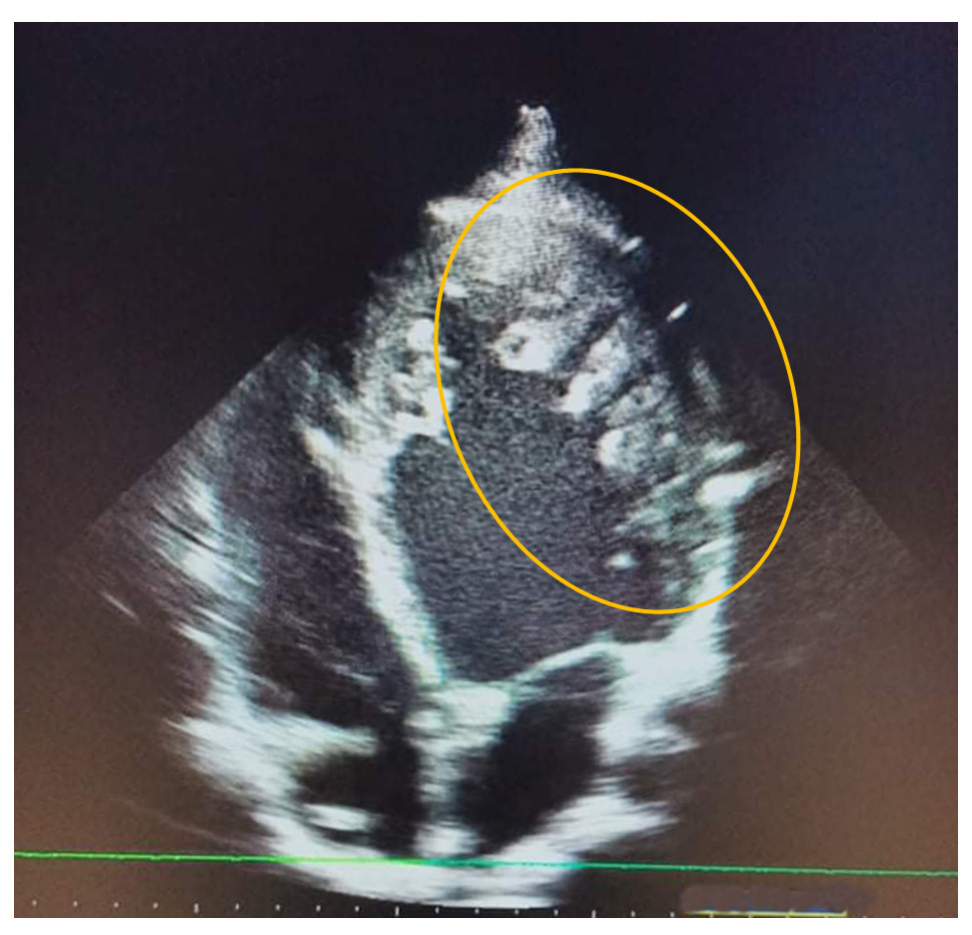
Transthoracic echocardiography aspect of the left free wall showing non compacted trabeculation (circle).

A 20- year -old male, who presented with dyspnea in March 2019, was followed up by the cardiology department and classified as stage 2 on the New York Heart Association (NYHA) Functional Classification. The patient had no other relevant personal or family past medical history. As part of an etiological assessment of dyspnea, a transthoracic echocardiography was requested showing a dilated left ventricle, reduced left ventricular ejection fraction at 40%, septo-apical myocardial hypokinesia and left ventricular hypertrabeculation ( Figure 1).

**Figure 2.**
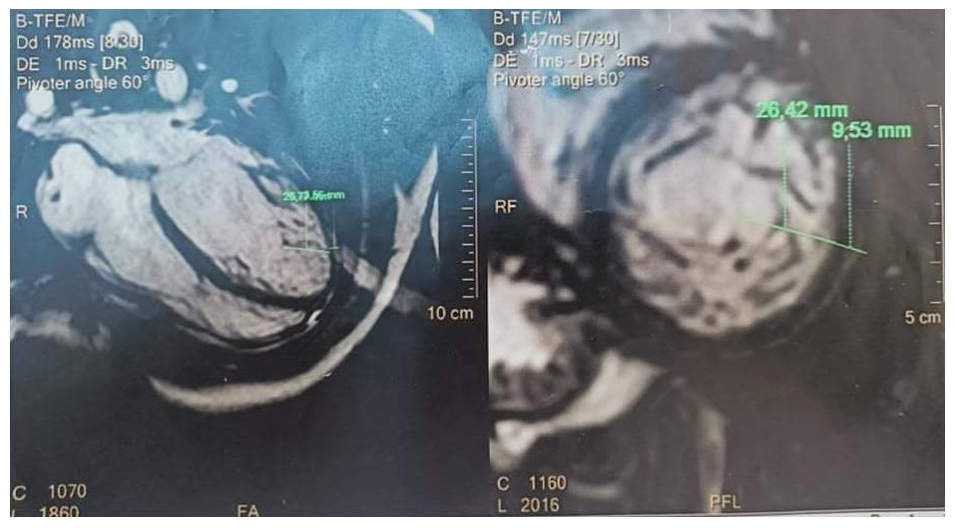
Cardiac magnetic resonance imaging showing non compacted trabeculation.

The diagnosis made was a heart failure, and patient was put under treatment. A cardiac magnetic resonance imaging (CMRI) was requested and revealed reduced left ventricular ejection fraction (LVEF) at 30%, globular shape of the left ventricle with an overall wall hypokinesia, hypertrabeculation located at the left ventricle, a thickness of 2.8cm of the trabeculated myocardium on the compact myocardium, less contrast enhancement of the trabeculated myocardium compared to normal, and no thrombus in the left ventricle ( Figure 2). It was concluded that the appearance was most likely left VNC cardiomyopathy with a LVEF at 30% without intra-cardiac thrombus or mitral insufficiency Figure 2.

The patient was later lost to follow-up. A few months later, he died without a clear cause explaining the death. A forensic autopsy was performed.

### Autopsy findings

The body was that of a man of average build, with an approximate weight of 70kg. There was no specific sign on the external examination particularly, no asymmetry in comparing the circumference of the two calves.

**Figure 3.**
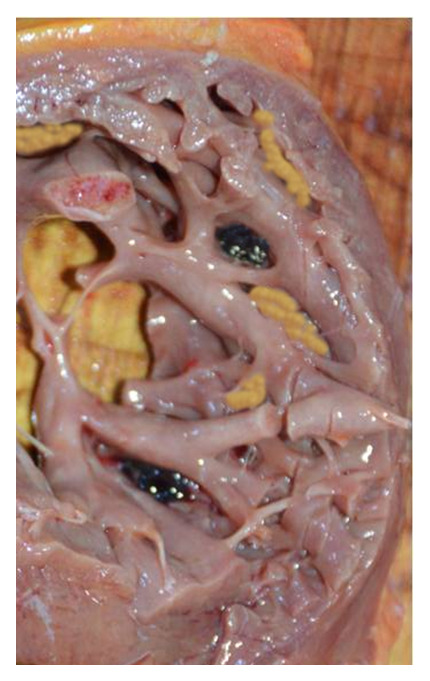
Necropsy aspect of the left ventricular wall revealed at autopsy.

The heart was globose weighing 325g. The coronary arteries were in a normal position without any significant lesion. The axial dissection of the heart found left ventricle hypertrabeculation with parietal thinning measured at 0.5cm and some intra-cavitary adhering thrombi Figure 3. Left ventricle examination found wall thickening at 1.2cm. Valvular examination was normal. No systemic thrombi was found. The lungs were the site of profuse oedema. The rest of the organs were congestive without any abnormality. Toxicology was negative. Based on clinical history and the necropsy findings, death was attributed to acute ventricle arrhythmia secondary to a myocardium non-compaction.

## Discussion

The European Society of Cardiology (ESC) categorize VNC as “unclassified cardiomyopathy” with a structural and functional abnormal heart muscle without any other diseases sufficient to cause the observed myocardial abnormality”. However, The American Heart Association (AHA) categorize it as “genetic cardiomyopathy” ^4^. The etiology and embryogenetic mechanisms leading to VNC are still unknown and several hypothesis are suggested. The most frequent on is that hypertrabeculation may result from excessive trabeculae formation and/or a defect in the later compaction processes ^1, 5^.

VNC, and specifically on the left side, has been found in association with more than 40 mutated genes, which encode for several cell structures. The most common are MYH7, MYBPC3, TTN, at a rate of 71% ^6^. Some cases of VNC associated with congenital hemoglobinopathies have been described in the literature.

Although the usual site of hypertrabeculation involvement is the left ventricle, the right ventricle is rarely affected. Right VNC can lead to ventricular tachycardia or right heart failure. In addition, patients with right VNC can be perfectly asymptomatic with only electrocardiographic disorder. In addition, concomitant damage of right VNC is not rare and it can be difficult to distinguish between non-compaction and arrhythmogenic right ventricular cardiomyopathy (ARVD). Diagnosis criteria for ARVD, even if it coexists with typical VNC, may lead to a diagnosis of ARVD rather than VNC. Less frequently, both ventricles can be affected leading to entirely non-compaction cardiomyopathy ^7^. 

Different imaging-based classification systems have been used to make VNC diagnosis. Cardiac Magnetic Resonance Imaging (CMRI)-based criteria (Petersen criteria) is considered as the gold standard ^8^. Not all definitions are anatomically controlled and these criteria are nonspecific. Autopsy performed on individuals with known VNC can be a good way to compare radiological criteria to anatomical findings. Collaboration between forensic medicine and cardiology should be take into consideration with the aim to standardize diagnostic criteria and to avoid over diagnosis in healthy people with a benign prognosis.

Nonspecific histopathological findings have been described, including hypertrophy of the cardiomyocytes, ischemic necrosis with fibrosis due to insufficient vascular supply of the trabeculations, and disorganization of cardiomyocytes ^9^. VNC leads to variable complications that can be misdiagnosed and at the origin of sudden cardiac death. The most common is conduction defects in approximately 90% of patients followed by myocardium arrhythmia. Thromboembolic events are not very frequent and occur in only 10% of cases, mostly in adults. Three main factors are involved in the occurrence of thromboembolic events: the presence of thrombi into ventricular trabeculations, left ventricular systolic dysfunction with reduced ejection fraction, and/or atrial fibrillation.

Currently, there are no guidelines for the management of patients with VNC. Recommendations for treatment include prophylactic anticoagulation therapy and the implantation of a cardiac defibrillator. Treatment for VNC is therefore that of any cardiomyopathy with heart failure ^10^. A periodic check with a 24-hour ECG holter is indicated in order to assess the risk of a possible asymptomatic arrhythmia. Finally, first-degree family members of all patients diagnosed with VNC should undergo an echocardiographic screening examination and genetic exploration ^11^.

In summary, there are multiple controversies related with VNC comprising etiology and pathogenesis, genetic findings, relation with extra-cardiac diseases, diagnostic criteria, treatment, and prognosis. Cardiologists have to pay attention to various clinical manifestations, including heart failure, arrhythmias and cardio embolic events, which can be related to VNC in order to initiate an early treatment and avoiding potentially fatal complications.

## Data availability

All data underlying the results are available as part of the article and no additional source data are required.

## Consent

Written informed consent for publication was obtained from the legally authorized representative of the decedent.

## References

[1] Choquet C Kelly RG Miquerol L : Defects in Trabecular Development Contribute to Left Ventricular Noncompaction. *Pediatr Cardiol.* 2019;40(7):1331–8. 10.1007/s00246-019-02161-9 31342111

[2] Kido K Guglin M : Anticoagulation Therapy in Specific Cardiomyopathies: Isolated Left Ventricular Noncompaction and Peripartum Cardiomyopathy. *J Cardiovasc Pharmacol Ther.* 2019;24(1):31–6. 10.1177/1074248418783745 29911432

[3] Stöllberger C Finsterer J : Understanding left ventricular hypertrabeculation/noncompaction: pathomorphologic findings and prognostic impact of neuromuscular comorbidities. *Expert Rev Cardiovasc Ther.* 2019;17(2):95–109. 10.1080/14779072.2019.1561280 30570401

[4] Loria V Colizzi C Vaccarella M : Left Ventricular Noncompaction: Cause or Consequence of Myocardial Disease? A Case Report and Literature Review. *Cardiology.* 2019;143(3–4):100–4. 10.1159/000500904 31509846

[5] Kayvanpour E Sedaghat-Hamedani F Gi WT : Clinical and genetic insights into non-compaction: a meta-analysis and systematic review on 7598 individuals. *Clin Res Cardiol.* 2019;108(11):1297–308. 10.1007/s00392-019-01465-3 30980206

[6] Bermudez-Jiménez FJ Jiménez-Jáimez J : Genotype, Family History, and Outcomes in Noncompaction Cardiomyopathy. *J Am Coll Cardiol.* 2018;71(24):2864. 10.1016/j.jacc.2018.03.527 29903360

[7] Luckie M Irwin B Nair S : Left ventricular non-compaction in identical twins with thalassaemia and cardiac iron overload. *Eur J Echocardiogr.* 2009;10(4):509–12. 10.1093/ejechocard/jen319 19091793

[8] Stöllberger C Finsterer J : Pitfalls in the diagnosis of left ventricular hypertrabeculation/non- compaction. *Postgrad Med J.* 2006;82(972):679–83. 10.1136/pgmj.2006.046169 17068279 PMC2653912

[9] Rambhatla T Mountantonakis S Bhasin K : Ventricular tachycardia due to isolated non-compaction of the right ventricle. *Eur Heart J Cardiovasc Imaging.* 2018;19(8):878. 10.1093/ehjci/jey068 29757364

[10] Gomathi SB Makadia N Ajit SM : An unusual case of isolated non-compacted right ventricular myocardium. *Eur J Echocardiogr.* 2008;9(3):424–5. 10.1093/ejechocard/jen016 18296401

[11] Aggarwal S Kalavakunta J Gupta V : A case of isolated right ventricle noncompaction with ST-Elevation chest leads. *Hear Views.* 2016;17(1):30. 10.4103/1995-705X.182645 27293528 PMC4879803

