# Supplementary material for: Case Report: Sudden cardiac death due to ventricular myocardial non-compaction
Source: F1000Res. 2021 Jul 29;9:1045. Originally published 2020 Aug 26. [Version 2] doi: 10.12688/f1000research.24583.2 (PMC8329599; doi:10.12688/f1000research.24583.2)
Supplement: Peer review history

# Peer review: Reviewer response for version 2

**DOI:** 10.5256/f1000research.58509.r90733

**Published:** 2021-08-02

**Authors:** Citrawati Dyah Kencono Wungu

**Affiliations:** 1 Department of Physiology and Medical Biochemistry, Faculty of Medicine, Universitas Airlangga, Surabaya, Indonesia; 2 Institute of Tropical Disease, Universitas Airlangga, Surabaya, Indonesia

The authors have made significant revisions as suggested. However, they should check again for grammatical errors, e.g:

- "Caucasian student male" should be "male Caucasian student".
- BMI was...
- "...septo-apical myocardial hypokinesia and left ventricular hypertrabeculation" should be "septo-apical myocardial hypokinesia, and left ventricular hypertrabeculation".
- "bisoprolol 2,5 mg per day and Ramipril..." should be "bisoprolol 2,5 mg per day, and Ramipril..."
- "death was attributed" should be "the death was attributed".

It will also be more comprehensive if histological figures are provided.

Are enough details provided of any physical examination and diagnostic tests, treatment given and outcomes?

No

Is the case presented with sufficient detail to be useful for other practitioners?

Partly

Is sufficient discussion included of the importance of the findings and their relevance to future understanding of disease processes, diagnosis or treatment?

Partly

Is the background of the case’s history and progression described in sufficient detail?

Partly

Reviewer Expertise:

Molecular cardiology

I confirm that I have read this submission and believe that I have an appropriate level of expertise to confirm that it is of an acceptable scientific standard.

# Peer review: Reviewer response for version 1

**DOI:** 10.5256/f1000research.27119.r88980

**Published:** 2021-07-05

**Authors:** Citrawati Dyah Kencono Wungu

**Affiliations:** 1 Department of Physiology and Medical Biochemistry, Faculty of Medicine, Universitas Airlangga, Surabaya, Indonesia; 2 Institute of Tropical Disease, Universitas Airlangga, Surabaya, Indonesia

In this case report, Dr. Mannoubi and colleagues reported a case of a man with ventricular non-compaction and sudden cardiac death. This case shows a good collaboration between cardiologists and forensics. However, I have some major and minor comments to improve the quality of the article, especially regarding the completeness of the data:

1. Several data are lacking about the patient, such as: the patient’s race (this is important due to the influence of genetic factors); how long the symptoms have occurred; symptoms other than dyspnea; the patient’s occupation; medications that have been taken; physical examination; laboratory findings; and how the patient was diagnosed with heart failure. Is there any cardiomegaly? Was the patient in shock at his admission to the hospital? ECG data is also important because ventricular non-compaction is closely related to arrhythmia.
2. Histology is present in the Abstract but no histopathological findings mentioned. The histopathological findings should be added.
3. Data regarding therapy administered to the patient is also not mentioned.
4. How much is the ratio of non-compacted myocardium to compact myocardium at the end of systole/diastole (Petersen criteria)?
5. Completing the patient’s data is important for the diagnosis of LVNC, as hypertrabeculation can also be observed in other comorbidities or even athletes. Will these conditions give different results in the autopsy findings? This should also be mentioned in the Discussion.
6. As the death of the patient was attributed to acute ventricle arrhythmia secondary to a myocardium non-compaction, the prognosis and mortality of the patients with LVNC should also be explained, like the mortality/survival rate.
7. “Some cases of VNC associated with congenital hemoglobinopathies have been described in the literature.” Please put citations and more explanations regarding this.
8. In the Discussion, please explain ECG findings that should be found in VNC.
9. “VNC leads to variable complications that can be misdiagnosed and at the origin of sudden cardiac death. The most common is conduction defects in approximately 90% of patients followed by myocardium arrhythmia. Thromboembolic events are not very frequent and occur in only 10% of cases, mostly in adults. Three main factors are involved in the occurrence of thromboembolic events: the presence of thrombi into ventricular trabeculations, left ventricular systolic dysfunction with reduced ejection fraction, and/or atrial fibrillation.”: Please put references to these statements.
10. The role of autopsy and collaboration between cardiologists and forensics should be added in the summary.
11. There are many grammatical errors, for example:
  - “Ventricular non-compaction (VNC) is a rare myocardium disorder…” should be “myocardial disorder”.
  - “...a transthoracic echocardiography was requested showing”. The word "requested" is unneeded.
  - “...and patient was put under treatment” should be “and the patient”.
  - “A cardiac magnetic resonance imaging (CMRI) was requested”, "requested" should be changed to "performed".
  - “ Left ventricle examination” should be “left ventricular”.
  - “Toxicology was negative” should be "toxicology examination" or "toxicology test".
  - “The European Society of Cardiology (ESC) categorize VNC” should be "categorizes".
  - “However, The American Heart Association (AHA) categorize” should be "categorizes".
  - “The most frequent on” should be “the most frequent hypothesis”.
  - “...at the origin of sudden cardiac death” should be “lead to sudden cardiac death”.
  -  “...in order to initiate an early treatment and avoiding” should be "avoid".

Are enough details provided of any physical examination and diagnostic tests, treatment given and outcomes?

No

Is the case presented with sufficient detail to be useful for other practitioners?

Partly

Is sufficient discussion included of the importance of the findings and their relevance to future understanding of disease processes, diagnosis or treatment?

Partly

Is the background of the case’s history and progression described in sufficient detail?

Partly

Reviewer Expertise:

Molecular cardiology

I confirm that I have read this submission and believe that I have an appropriate level of expertise to confirm that it is of an acceptable scientific standard, however I have significant reservations, as outlined above.

# Response

**Published:** 2021-07-07

**Authors:** med amin mesrati

**Affiliations:** Taher Sfar Hospital, Tunisia

Dear Reviewer,

 First, we thank you for the interest and the time that you have gave to the article. This manuscript was revised according to your relevant comments. We realize that the quality of the article is now improved.

 We hope that these changes make our work acceptable for indexing.

 Best regards,

# Peer review: Reviewer response for version 1

**DOI:** 10.5256/f1000research.27119.r70295

**Published:** 2020-09-09

**Authors:** Mokhles lajmi

**Affiliations:** 1 Department of Cardiac and Thoracic Surgery, The Military Hospital of Tunis, Tunis, Tunisia

This is a case report about a young male deceased from ventricular arrhythmia due to left ventricular non compaction (VNC) syndrome, a rare but quite controversial pathology.

 In fact, this condition can express itself in various ways (ranging from completely asymptomatic patients to severe heart failure or arrhythmias) misleading the diagnosis, and imagery criteria are lacking specificity.

 We thank the authors for this quality case report.

**Q1:**

- The case description had all the necessary information to fully understand the case.

**Q2:**

- Clinical findings (autopsy findings in this case) are accurate and concise.

**Q3:**

- I congratulate the author's for their interesting and insightful point of view regarding the potential importance of collaboration between forensics and cardiology to further improve the diagnosis accuracy. In fact diagnosis of VNC is very controversial and misleading, and imaging criteria are perplexing, thus dealing with VNC from a cardiology-only point of view is narrow minded, forensics could be a huge asset to correlate imagery to anatomy.
- We know that some degree of non compaction of the left ventricle is not uncommon in the anterior apical region and trabeculations of the left ventricle can be increased in physiological conditions such as pregnancy and exercise training, would this be a diagnostical challenge for forensics? Can this mislead the autopsy findings?  Is there any differential diagnosis in VNC?

**Q4:**

- Author's succeeded in explaining a relatively complex pathology, in a way that it's understandable for non cardiologists/forensics.

**5: **The author's should consider the following points:

- In accordance with CARE guidelines for case reports, "case report" should appear in keywords, with no more than 5 keywords
- As mentioned in the introduction, ventricular non compaction was first described in 1926. I think it would be interesting to add that it was done by Mr Grant and cite the reference:
  Grant RT. An unusual anomaly of the coronary vessels in the malformed heart of a child. Heart 1926; 13: 273–83. ^1^
- Regarding the prevalence of this disease, although it was deemed rare by many investigators, the distribution of this disease in the global population is very difficult to evaluate, and numbers diverge hugely in the literature. One referenced source in a Lancet paper even stated that it “seems to be the third most commonly diagnosed cardiomyopathy”. ^2^ Thus I advise to not mention a particular prevalence and just state that it is rare and that its prevalence is uncertain.
- Figure 2: it would be more accurate to mention: non compacted left trabeculations.
- Discussion section: Some cases of VNC associated with congenital hemoglobinopathies have been described in the literature. Author's should add a reference relating to this fact. ^3^^,^^4^
- VNC abbreviation is used in abstract. Abstracts should not contain abbreviations unless it is necessary.

6/ We noted some spelling errors: 

- Autopsy findings, line 5; change "found wall thickening at 1.2cm" to " found a wall thickening of 1.2cm".
- Autopsy findings, line 7; "abnormalities" instead of ""abnormality".
- Discussion, line 12; "disorders" instead of "disorder".
- Discussion, line 12: "complications of right VNC are not rare " instead of "concomitant damage".
- Discussion, line 19: "taken" instead of "take".

Are enough details provided of any physical examination and diagnostic tests, treatment given and outcomes?

Yes

Is the case presented with sufficient detail to be useful for other practitioners?

Yes

Is sufficient discussion included of the importance of the findings and their relevance to future understanding of disease processes, diagnosis or treatment?

Yes

Is the background of the case’s history and progression described in sufficient detail?

Yes

Reviewer Expertise:

cardiovascular surgery

I confirm that I have read this submission and believe that I have an appropriate level of expertise to confirm that it is of an acceptable scientific standard.

## References

[1] : An unusual anomaly of the coronary vessels in the malformed heart of a child. *Heart* .1926;13:273-283

[2] : Left ventricular non-compaction cardiomyopathy. *Lancet* .2015;386(9995) : 10.1016/S0140-6736(14)61282-4 813-25 10.1016/S0140-6736(14)61282-4 25865865

[3] : Non-compaction cardiomyopathy. *Forensic Sci Med Pathol* .2016;12(1) : 10.1007/s12024-016-9743-7 104-7 10.1007/s12024-016-9743-7 26803832

[4] : Left ventricular hypertrabeculation/noncompaction. *J Am Soc Echocardiogr* .2004;17(1) : 10.1016/S0894-7317(03)00514-5 91-100 10.1016/S0894-7317(03)00514-5 14712181

# Response

**Published:** 2020-09-11

**Authors:** med amin mesrati

**Affiliations:** Taher Sfar Hospital, Tunisia

Q1: The case description had all the necessary information to fully understand the case.

*No comments*

 Q2: Clinical findings (autopsy findings in this case) are accurate and concise.

*No comments*

 Q3: I congratulate the author's for their interesting and insightful point of view regarding the potential importance of collaboration between forensics and cardiology to further improve the diagnosis accuracy. In fact diagnosis of VNC is very controversial and misleading, and imaging criteria are perplexing, thus dealing with VNC from a cardiology-only point of view is narrow minded, forensics could be a huge asset to correlate imagery to anatomy.

 We know that some degree of non compaction of the left ventricle is not uncommon in the anterior apical region and trabeculations of the left ventricle can be increased in physiological conditions such as pregnancy and exercise training, would this be a diagnostical challenge for forensics? Can this mislead the autopsy findings?  Is there any differential diagnosis in VNC?

*R: After excluding dilated and hypertrophic cardiomyopathy, forensic doctor should consider other diagnoses that can present with similar features. Thus, it is essential to distinguish left ventricular non-compaction from acquired changes seen in pulmonary atresia, mycotic invasion of the heart… and literature disclosed many other differential diagnoses. In our context, the MRI helped us to retain VNC as final diagnosis.*

 Q4: Author's succeeded in explaining a relatively complex pathology, in a way that it's understandable for non cardiologists/forensics.

*No comments*

 5: The author's should consider the following points:

 In accordance with CARE guidelines for case reports, "case report" should appear in keywords, with no more than 5 keywords

*R: it is considered*

 As mentioned in the introduction, ventricular non compaction was first described in 1926. I think it would be interesting to add that it was done by Mr Grant and cite the reference:

 Grant RT. An unusual anomaly of the coronary vessels in the malformed heart of a child. Heart 1926; 13: 273–83.1

*R: it is considered*

 Regarding the prevalence of this disease, although it was deemed rare by many investigators, the distribution of this disease in the global population is very difficult to evaluate, and numbers diverge hugely in the literature. One referenced source in a Lancet paper even stated that it “seems to be the third most commonly diagnosed cardiomyopathy”.2 Thus I advise to not mention a particular prevalence and just state that it is rare and that its prevalence is uncertain.

*R: it is considered*

 Figure 2: it would be more accurate to mention: non compacted left trabeculations.

*R: It is considered*

 Discussion section: Some cases of VNC associated with congenital hemoglobinopathies have been described in the literature. Author's should add a reference relating to this fact.

 VNC abbreviation is used in abstract. Abstracts should not contain abbreviations unless it is necessary.

*R: it is considered*

 6/ We noted some spelling errors:

 Autopsy findings, line 5; change "found wall thickening at 1.2cm" to " found a wall thickening of 1.2cm".

 Autopsy findings, line 7; "abnormalities" instead of ""abnormality".

 Discussion, line 12; "disorders" instead of "disorder".

 Discussion, line 12: "complications of right VNC are not rare " instead of "concomitant damage".

 Discussion, line 19: "taken" instead of "take".

*R: considered*
